# Crossing the street in front of an autonomous vehicle: An investigation of eye contact between drivengers and vulnerable road users

**DOI:** 10.3389/fpsyg.2022.981666

**Published:** 2022-10-28

**Authors:** Aïsha Sahaï, Elodie Labeye, Loïc Caroux, Céline Lemercier

**Affiliations:** CLLE, UMR 5263, Centre National de la Recherche Scientifique (CNRS), Toulouse Jean Jaurès University, Toulouse, France

**Keywords:** road safety, pedestrians, cyclists, kick scooter users, autonomous vehicles, eye contact, human-machine interface

## Abstract

Communication between road users is a major key to coordinate movement and increase roadway safety. The aim of this work was to grasp how pedestrians (Experiment A), cyclists (Experiment B), and kick scooter users (Experiment C) sought to visually communicate with drivengers when they would face autonomous vehicles (AVs). In each experiment, participants (*n* = 462, *n* = 279, and *n* = 202, respectively) were asked to imagine themselves in described situations of encounters between a specific type of vulnerable road user (e.g., pedestrian) and a human driver in an approaching car. The human driver state and the communicative means of the approaching car through an external Human-Machine Interface (eHMI) were manipulated between the scenarios. The participants were prompted to rate from “never” to “always” (6-point Likert scale) the frequency with which they would seek eye contact with the human driver either in order to express their willingness to cross or to make their effective decision to cross. Our findings revealed that a passive human driver in an AV with no visual checking on the road triggered a decline in vulnerable road users’ desire to communicate by eye contact (Experiments A–C). Moreover, the results of Experiment C demonstrated that the speed screen, the text message screen, and the vibrating mobile app eHMI signals diminished kick scooter users’ desire to communicate visually with the human driver, with some age-based differences. This suggested a better comprehension of the approaching car’s intentions by the kick scooter users, driven by the features of the eHMI.

## Introduction

The road environment included various types of users who must interact in a safe and optimal way in order to ensure traffic fluency. Among these, vulnerable road users, that is to say, users with a poor level of body protection in the event of a collision with a four-wheeled motor vehicle (e.g., pedestrians, cyclists, motorcyclists, and kick scooter users) represented 45% of the reported fatalities in France in 2020 (ONISR, 2021, p. 14). More specifically, the French 2019 road safety annual report (ONISR, 2020, p. 62) reported a slight increase in pedestrian fatalities in individuals aged 18–34 years, followed by those aged 35–64 and 65–74 years, and a major peak in individuals aged 75 and more. Concerning cyclists, a 2011 report on cyclist accidents in France (CEREMA, 2018, p. 12) highlighted some clear rises in fatalities among individuals aged 11–25 years and among those aged 46–80 years. Concerning kick scooter users, Bagou et al. (2021) reported a major fatality peak in individuals aged 20–24 years, followed by high rates in individuals aged 10–19 years and in those aged 25–34 years (Bagou et al., 2021). Taken together, these differences in fatal injury occurrences across vulnerable road users suggest specificities in the way they behave in the road environment.

With the forthcoming fleet of autonomous vehicles (AVs) in road traffic, vulnerable road users may become even more at risk as these vehicles’ intentions may not be properly understood (Liu H. et al., 2020). Hence, a misunderstanding of AVs’ intentions could generate a feeling of uncertainty among vulnerable road users (Lagström and Lundgren, 2015; Palmeiro et al., 2018) and downright unsafe decision-making. Specifically, at level-3 and level-4 of driving automation, the human behind the steering wheel inside the AV is not required to monitor the driving task or to look at the road when the automated driving system is turned on (SAE, 2018). Hence, the human driver inside a level-3 or level-4 AV can sometimes be a passenger, which led authors to coin the term “drivenger” (a contraction of “driver” and “passenger”) to label the occupant of a level-3 or level-4 AV (Reilhac et al., 2015; Lemercier et al., 2021). Moreover, in a level-3 or level-4 AV, the drivenger is likely to carry out non-driving-related tasks (e.g., reading, gaming, working, etc.) and be impeded from sending explicit communication cues to vulnerable road users (and all other road users). This is even more the case with level-5 of automation, where the human driver is intended to be merely absent, for the purpose of transporting passengers or goods. Consequently, the decline in communication between the human driver in the AV and the external road users may lead the latter to behave inappropriately due to false assumptions about the AV’s intentions. In this context, the aim of the present study was to grasp how vulnerable road users would seek to visually communicate with drivengers when facing AVs.

Indeed, it has been highlighted that road users are likely to use informal non-verbal communication cues in order to express and attribute intentions, in particular during complex and confusing situations (Rasouli et al., 2017; Sucha et al., 2017; Liu Y. et al., 2020; Bazilinskyy et al., 2022). For example, by analyzing 650 video clips of real traffic scenes in different countries, Rasouli et al. (2017) found that gaze was the most prominent form of non-verbal communication cue used by pedestrians in order to transmit a crossing intention to an upcoming car driver. Interestingly, the authors reported that older adult pedestrians’ looks prior to crossing were on average one second longer than those of children and adults, suggesting some age-based disparities in pedestrians’ visual behavior. Moreover, based on on-site observations and interviews, Sucha et al. (2017) reported that in addition to the yielding behavior of oncoming vehicles, pedestrians considered drivers’ gestures and the establishment of eye contact with the drivers as signs that the drivers of the vehicles would stop in order to let them cross the street at a crosswalk. Consistently, based on focus group interviews, Liu Y. et al. (2020) reported that pedestrians sought communication with vehicle drivers, especially on unsignalized roads, or when vehicles are on standby, turning or reversing, or when priority rules are not clear. With regard to cyclists, Bazilinskyy et al. (2022) revealed through a scenario-based immersion experiment that blinded windows of an approaching car at an intersection increased the cyclists’ willingness to brake when crossing, while detectable driver eye contact caused them to want to continue pedaling. There is therefore a body of evidence suggesting that road users feel the need to make eye contact with drivers before making their crossing decisions, most likely to remove the ambiguity about who has the right of way and make safer crossing decisions. In other words, it seems that eye contact provides the foundations of communication and social interactions between road users (Senju and Johnson, 2009; Jording et al., 2019).

Considering the foreseeable lack of eye contact and other forms of non-verbal communication from the occupants of AVs, the use of external Human-Machine Interfaces (eHMIs) has been proposed both by manufacturers (e.g., Mercedes-Benz, 2015; Mitsubishi Electric, 2015; Nissan Motor Corporation, 2015; Semcon, 2016; Daimler, 2017) and academics (e.g., Clamann et al., 2017; de Clercq et al., 2019; Kaleefathullah et al., 2020; Dey et al., 2021) as a means to send explicit communication signals to the surrounding road users, thereby mitigating the uncertainty about the AV’s intention or state. The suggested eHMIs have been designed in various ways, ranging from text-based screens or light signals placed on the AV to projectors located on the AV and that project light signs on the road, and also personal wearable devices connected to the AV (see Bazilinskyy et al., 2019; Rasouli and Tsotsos, 2019 for reviews).

For example, de Clercq et al. (2019) asked pedestrian participants to assess their level of safety when considering crossing in front of a yielding virtualized AV that exhibited an eHMI in the form of colored front brake lights or a screen attached outside the vehicle and displaying either a knightrider animation, a text message stating “walk,” or a smiley. In the control condition, the AV was not fitted with an eHMI. The authors reported that participants’ felt safer crossing in front of AVs fitted with an eHMI compared to no eHMI, and no differences were found between the different sorts of eHMIs. However, Kaleefathullah et al. (2020) pointed out that a learning curve was necessary for pedestrian participants to understand the meaning of abstract signals such as light-based signals when they faced such a kind of eHMI message for the first time.

Furthermore, Holländer et al. (2020) showed that the use of smartphone apps that display traffic guidance information (e.g., showing a green or red light depending on whether it is safe to cross or not or a red or green border on the left or right side of the user’s smartphone screen according to the approach side of the coming vehicle, or otherwise showing the user’s position together with the location of nearby vehicles) coupled with vibrotactile signals, enhanced pedestrian participants’ willingness to cross in videos of traffic scenarios, when compared with no guidance (Holländer et al., 2020). In the same vein, Cœugnet et al. (2017) demonstrated that the use of a vibrotactile wristband that emitted a vibration when the forthcoming crossing was judged to be unsafe helped to decrease the percentage of pedestrian decisions that led to collisions with an approaching car in a virtual reality environment (Cœugnet et al., 2017).

Lastly, in a real-world study, Clamann et al. (2017) asked pedestrian participants to cross in front of an AV for which the eHMI consisted of a screen attached outside the vehicle that could display either the actual speed of the AV or an advice to cross in the form of a pictogram showing a pedestrian. In two other control conditions, the eHMI was turned off or absent. The results did not show a benefit of one or the other eHMI messages on the participants’ decision time to cross with regard to the control conditions. Nevertheless, presenting the speed of the AV could still be seen as an interesting approach as this piece of information is universal and not targeted to one particular road user, contrary to crossing advice. Indeed, the vehicle speed cue can be used simultaneously by a variety of individuals who coexist at different locations of the road traffic without being inaccurate. In addition, the median age of the participants in the study by Clamann et al. (2017) was quite low (*m* = 22), and it is possible that the young adult participants were already able to take effective crossing decisions as suggested by prior work (Dommes et al., 2012). However, older adult pedestrians would possibly have benefitted from this type of eHMI signal as difficulties in taking safe crossing decisions were highlighted for this age population (Dommes and Cavallo, 2011; Lobjois et al., 2013; Petzoldt, 2014). Hence, communicating the vehicle speed information to the older adult pedestrians could induce a sharper mental model (Johnson-Laird, 2005) of the approaching car’s kinematics in these road user’s cognitive systems. In turn, this accurate mental model of the vehicle behavior could induce better anticipatory behavior such as earlier road entrance or walking speed acceleration when possible, for example. It should also be mentioned that some investigations showed that the implicit cues embodied in the vehicle’s behavior (e.g., its approaching speed and relative distance) were enough to grasp the intention of the AV as pedestrians mostly relied on this sort of information in order to make their crossing decisions (Clamann et al., 2017; Palmeiro et al., 2018; Dey et al., 2021; Lee et al., 2021). Yet, explicit communication from AVs to vulnerable road users could still be regarded as a key component in addressing uncertainties in ambiguous situations although there is no consensus to date about the best eHMI signal to deliver (Merat et al., 2018; de Clercq et al., 2019).

Another factor to consider when dealing with the communication from AVs to other road users is the degree of attention of the drivenger (when not absent) toward the road environment. Indeed, strong links have been made between the level of driving automation and the drivengers’ propensity to monitor the road and/or the driving task (Navarro, 2019; Navarro et al., 2019). Moreover, it has been highlighted that a high level of trust in automation could lower drivengers’ road-directed gaze behavior during automated driving (Hergeth et al., 2016; Körber et al., 2018; Walker et al., 2018). Accordingly, it could be said that variable visual attentional states can be attributed to drivengers, which are not inconsequential on the other road users’ behavior. For example, Lagström and Lundgren (2015) found that pedestrian participants’ willingness to cross in front of a vehicle was enhanced when the participants were able to make eye contact with an attentive driver as compared to a distracted driver talking on the phone or reading a newspaper. Furthermore, the participants in this study reported a more negative experience, interpreted as distress, when imagining themselves crossing in front of a vehicle in which the driver was reading a newspaper compared to talking on the phone, with both being worse than when the driver was attentive to the road with her hands on the steering wheel (Lagström and Lundgren, 2015). Interestingly, Faas et al. (2021) showed that pedestrians’ degraded perception of safety in front of inattentive drivers could be mitigated with the help of an eHMI (Faas et al., 2021). In the authors’ study, the use of an eHMI in the form of continuously lit strip bands placed on the AV sufficed to reinforce the participants’ perceived safety when they had to cross in front of a drivenger reading a newspaper or even a tinted-windshield-AV. Thus, it has been argued that such a kind of eHMI could help pedestrian participants to ignore the state of the driver if they realized they were facing an AV (Faas et al., 2021). Consistently, in their scenario-based immersion experiment, Bazilinskyy et al. (2022) showed that when cyclists were about to cross in front of an AV with a inattentive driver who was texting inside, they were likely to want to keep pedaling if the AV was exhibiting a “GO” message on a screen eHMI in contrast to no eHMI device. A similar pattern of results was observed in the authors’ study (Bazilinskyy et al., 2022) when the AV had tinted windows, i.e., when the drivenger was no longer visible. Hence, these results suggested removal of uncertainty among cyclists, driven by the text message crossing advice exhibited by the AV. Taken all together, it appears that vulnerable road users might benefit from eHMI signals when interpersonal visual communication between road users is ruptured.

However, little is known about how vulnerable road users would react given a multiplicity of eHMI messages when facing AVs. Besides, most of the previous investigations focused on pedestrians whereas cyclists are becoming increasingly present in urban road traffic due to the health and cost benefits of cycling (WHO, 2021). At the same time, kick scooters are becoming a popular means of transportation among young road users in urban areas due to their electrification and their growing availability for free-service rental (e.g., free-service kick scooter services from Lime, Bird, and Circ, in Paris). Yet, hardly any attention has been given to kick scooter users in scientific research when addressing communication between AVs and vulnerable road users. Considering these elements, three survey questionnaire experiments were conducted with the aim of exploring how visual communication would be issued by pedestrians (Experiment A), by cyclists (Experiment B), and by kick scooter users (Experiment C) when they face AVs. In particular, we aimed at exploring how eye contact would be used by pedestrians to (a) express their willingness to cross and (b) take their effective decision to cross when facing more or less attentive visible drivengers in AVs that could be equipped with different types of eHMIs. Questionnaires were used as they made it possible to target a large sample of participants. Grasping how vulnerable road users seek to visually communicate with drivengers can help prevent misunderstanding, and consequently, may help reduce incidents and accidents involving them.

Our first hypothesis (H1) was that the more the drivenger would be inattentive to the road environment, the less vulnerable road users (Experiments A–C) would envisage eye contact since if the drivenger is not looking at the road environment, interpersonal visual communication is forcibly stopped. Thus, when the drivenger would not be looking at the road, vulnerable road users would be reluctant to use the eye contact cue either to express their willingness to cross or to take their effective crossing decision.

Our second hypothesis (H2) was that the presence of an eHMI signal would reduce vulnerable road users’ willingness to envisage eye contact with the drivenger compared to the absence of an eHMI signal. Indeed, because the dispatched signal would express the vehicle’s state (e.g., the current speed) or intent (e.g., to yield or not), this would presumably mitigate uncertainty among vulnerable road users (Experiments A–C).

Moreover, one could surmise that the effect of the state of the human driver on eye contact communication would be modulated by the presence of the eHMI (H3), as suggested by previous work (Faas et al., 2021; Bazilinskyy et al., 2022). Hence, we predicted an interaction effect between these two variables with eye communication being lessened in the presence of an attentive drivenger when the approaching car exhibited an eHMI signal (Experiments A–C).

Finally, our fourth hypothesis was that vulnerable road users’ visual communication would vary across ages (H4). This part of our investigation was exploratory, however. Indeed, age-related differences in reported fatal injuries (CEREMA, 2018; ONISR, 2020, p. 62; Bagou et al., 2021, p. 12) appeared to suggest variations in the way pedestrians (Experiment A), cyclists (Experiment B), and kick scooter users (Experiment C) interact with other road users.

## Experiment A: Pedestrians

### Methods

#### Participants

Participants were recruited through social media and student networks. To be included in this survey questionnaire experiment, participants had to report walking as their most frequent mode of travel. Hence, 462 French-speaking participants took part in the experiment (312 women and 150 men; mean age = 43.25 years old; SD = 17.44 years; age range = 18–90). Among them, 69.61% were living in town whereas the others were living in the countryside; 54.74% of the participants reported walking every day, 31.03% reported walking several times a week, while 11.21 and 3.02% reported walking several times a month and several times a year, respectively.

#### Material

The questionnaire experiment was created using Qualtrics, an online survey construction tool. The first part of the questionnaire consisted of six queries regarding the participants’ demographics (e.g., gender, age) and travel habits (e.g., favorite mode of mobility, beginning of usage, travel frequency, and place of living). Then, the questionnaire proposed four scenarios describing situations of an encounter between a pedestrian and a human driver in an approaching car (see Supplementary Appendix A for an exhaustive description). All encounters took place in the absence of traffic lights and near a crosswalk enabling the person being on the pavement to cross a single lane. In addition to a written description of each situation of the encounter, a picture showing an approaching vehicle near a crosswalk was shown to the participants. Participants were told to imagine themselves as the pedestrian in each of the four scenarios, standing on the pavement with the intention to cross on the nearby crosswalk while a vehicle was approaching.

The state of the human driver located in the approaching car was manipulated across the four scenarios using a within-participant design. There were four driver states, namely, an active human driver in a traditional car (i.e., the human driver was in a traditional car and managed all the aspects of the driving task) as a control condition, an active human driver in an AV (i.e., the human driver was in an AV but managed all the aspects of the driving task), a passive human driver in an AV with visual checking (i.e., the human driver was in an AV and did not manage the driving task, but was still looking at the road), and a passive human driver in an AV with no visual checking (i.e., the human driver was in an AV and neither managed the driving task nor looked at the road). When the approaching car was an AV, the following description about the functioning of AVs was given: “The AV is able to manage the various aspects of the driving task such as lane keeping, acceleration and braking, and obstacle detection without assistance from the human driver inside the vehicle when the automated driving system is turned on.” Moreover, when the approaching vehicle was an AV, a sensor was depicted on the roof of the vehicle shown in the picture accompanying the written description, whereas when the approaching vehicle was a traditional car, there was no sensor on the vehicle in the picture.

**FIGURE 1:**
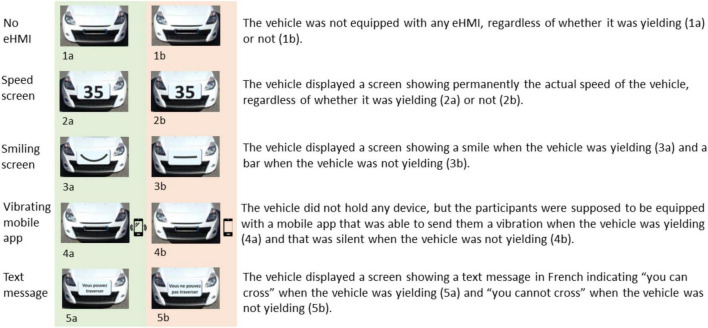
The different types of eHMI signals used in the experiment.

Furthermore, the type of eHMI displayed on the approaching car (regardless of whether it was a traditional car or an AV) was manipulated using a between-participants design so that each participant was presented with a single eHMI signal (see Figure 1). There were five possible eHMI conditions: no eHMI (i.e., the approaching car had no eHMI), a speed screen (i.e., the speed of the approaching car was displayed on a front screen located on the outside of the vehicle), a smiling screen (i.e., a smile was displayed on a front screen located on the outside of the vehicle when the approaching car was yielding, otherwise a horizontal bar was displayed on the screen), a vibrating mobile app (i.e., the vehicle did not display any device but a vibration was sent to the user through a mobile app when the approaching car was yielding), and a text message screen (i.e., a text message, written in French, indicating “you can cross” when the approaching car was yielding and “you cannot cross” otherwise, was displayed on a front screen located on the outside of the vehicle). These various eHMI signals were chosen because they each had a specificity that makes them relevant for real-life situations. For example, the speed screen eHMI display is a device that is not dependent on the position of the external road users. Hence, the information given by this kind of eHMI is true for those behind, in front of or beside the vehicle. Concerning the smiling screen, it endorsed an anthropomorphic view assumed to generate an intuitive understanding of the signal (Semcon, 2016). The interest of the vibrating mobile app is that it frees the visual channel of its users, leaving them available to collect other relevant information in the road environment, thereby spreading the user’s mental workload over different resource channels, and limiting performance breakdown (Wickens, 2008). Finally, the text message screen provided an explicit crossing advice that removed any ambiguity as to how to behave toward the approaching car (Fridman et al., 2019).

**FIGURE 2:**
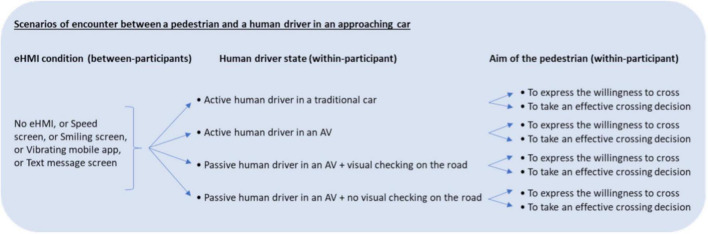
Independent variables manipulated in the four scenarios of encounters between a pedestrian and a human driver in an approaching car, with regard to the aim of the pedestrian.

For each scenario of an encounter between a pedestrian and a human driver in an approaching car (one specific eHMI signal and four human driver states), the participants had to rate on a 6-point Likert-type scale the frequency with which they would seek eye contact with the human driver (a) to express their willingness to cross to the human driver and (b) to take their effective crossing decision (see Figure 2). The endpoints of the Likert scale were “never” and “always,” and the intermediate points were “rarely,” “from time to time,” “sometimes,” and “often.” When the aim of the participant was to express a willingness to cross, the item to be rated was “you seek eye contact with the driver.” When the aim of the participant was to make an effective crossing decision, the item to be rated was “you ensure that the driver has made eye contact.” The entire questionnaire was written in French.

#### Procedure

A web link referring to the survey questionnaire was posted on different social media through public association groups. Once participants clicked on the link, they were presented with an overview of the study and were requested to give their informed digitized consent in order to pursue the experiment. Then, the questionnaire was displayed (see section “Material” for details). For each road user class, the eHMI condition was pseudo-randomly selected by the Qualtrics’ algorithm so that all eHMI conditions were equally dispatched between the participants.

#### Factorial plan and operational hypotheses

##### Independent variables

The first independent variable was the human driver state (with four modalities, namely, human driver driving a traditional car, human driver driving an AV, passive human driver in an AV with visual checking on the road, and passive human driver in an AV with no visual checking on the road), according to a within-participant design.

The second independent variable was the type of eHMI fitted on the approaching car (with five modalities, namely, no eHMI, speed screen, smiling screen, vibrating mobile app, and text message screen), according to a between-participant design.

Moreover, in order to investigate a possible age-related effect, the participants were assigned to one of four age groups, namely, “pedestrians under 35 years old,” “35- to 64-year-old pedestrians,” “65- to 74-year-old pedestrians,” and “pedestrians 75 years old or more,” according to their self-reported age. These age group intervals were defined with regard to French pedestrian accidentology data (ONISR, 2020, p. 62). The participant’s age group was considered in the following analyses according to a between-participant design.

##### Dependent variables

The dependent variable was the frequency (e.g., “never,” “rarely,” “from time to time,” “sometimes,” “often,” or “always”) with which the participants would consider eye contact with the human driver, either to express their willingness to cross, or to take their effective crossing decision. The participants’ raw responses were coded using integer numeric values from 0 to 5, assigning the value of “0” to “never” and “5” to “always.” The intermediate values were used for coding “rarely,” “from time to time,” “sometimes,” and “often.” Consequently, the frequency score with which the participants would consider eye contact with the human driver could vary on an ordinal scale from “0,” meaning that the cue was never used by the participants, to “5,” meaning that the cue was always used by the participants, with respect to their prior intention (e.g., to express their willingness to cross, or to make their crossing decision).

##### Operational hypotheses

We predicted that the participants’ frequency scores for seeking eye contact in order to express their willingness to cross or to take their crossing decision would be lower when:

– The human driver in the approaching car was passive in an AV with no visual checking on the road compared to when the human driver was passive in an AV with visual checking on the road, and compared to when the human driver was driving in an AV or in a traditional car, as a within-participant effect (H1);
– The participants received an eHMI signal compared to no eHMI signal, as a between-participants effect (H2);
– The human driver in the approaching car was actively driving when the participants were given an eHMI signal compared to when they received no eHMI signal (H3).

#### Statistical analyses

Statistical analyses were performed using R software. A mixed analysis of variance (ANOVA) was computed on the participants’ frequency scores for seeking eye contact with the human driver of the approaching car, either to express their willingness to cross, or to take their effective crossing decision. The type of human driver state (human driver driving a traditional car, human driver driving an AV, passive human driver in an AV with visual checking on the road, and passive human driver in an AV with no visual checking on the road) was considered as a within-factor in the mixed ANOVA. The type of eHMI (no eHMI, speed screen, smiling screen, vibrating mobile app, and text message screen) and the participants’ age group (under 35, 35–64, 65–74, and 75 years old or more) were considered as between-factors in the mixed ANOVA. In addition, *post-hoc* comparisons were made using the Wilcoxon signed-rank test for the within-factor and the Wilcoxon rank sum test for the between-factors, applying the False Discovery Rate (FDR) correction (Benjamini and Hochberg, 1995). The significance level was set at α = 0.05 for all tests.

### Results

#### Eye contact in order to express a willingness to cross

**FIGURE 3:**
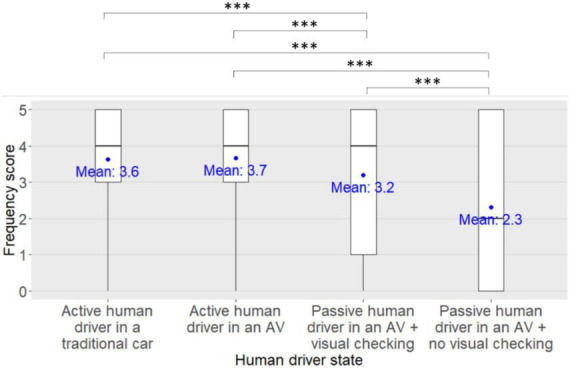
Main effect of the human driver state on the pedestrians’ frequency scores for seeking eye contact with the driver of the approaching car in order to express their willingness to cross to the human driver. ***: *pFDR* < 0.001.

Regarding the pedestrians’ frequency scores for seeking eye contact with the human driver of the approaching car in order to express their willingness to cross to the human driver, a significant main effect of the human driver state was found [*F*(3,1326) = 122.76, *p* < 0.001, η^2^*_*p*_* = 0.22, see Figure 3]. No main effect of the eHMI type [*F*(4,442) = 0.63, *p* = 0.64, η*^2^_*p*_* = 0.01] nor of the participant’s age group [*F*(3,442) = 1.99, *p* = 0.11, η*^2^_*p*_* = 0.01] were found. However, a significant human driver state x eHMI x participants’ age group interaction was found [*F*(36,1326) = 2.21, *p* < 0.001, η*^2^_*p*_* = 0.06] although it was not supported by the *post-hoc* analyses (all *pFDRs* > 0.05). No other significant interactions between factors were found (all *ps* > 0.05).

*Post-hoc* analyses investigating the main effect of the human driver state on the participants’ frequency scores for seeking eye contact with the human driver of the approaching car in order to express their willingness to cross to the human driver revealed that the participants’ frequency scores were significantly lower when the human driver was passive in an AV with no visual checking on the road compared to when the human driver was passive in an AV with visual checking on the road (*pFDR* < 0.001), and compared to when the human driver was driving in an AV (*pFDR* < 0.001) or in a traditional car (*pFDR* < 0.001). In addition, the participants’ frequency scores were significantly lower when the human driver was passive in an AV with visual checking on the road compared to when the human driver was driving in an AV (*pFDR* < 0.001) or in a traditional car (*pFDR* < 0.001). Finally, there was no difference in the participants’ frequency scores between when the human driver was driving in an AV and when the human driver was driving in a traditional car (*pFDR* = 0.18).

#### Eye contact in order to take an effective crossing decision

**FIGURE 4:**
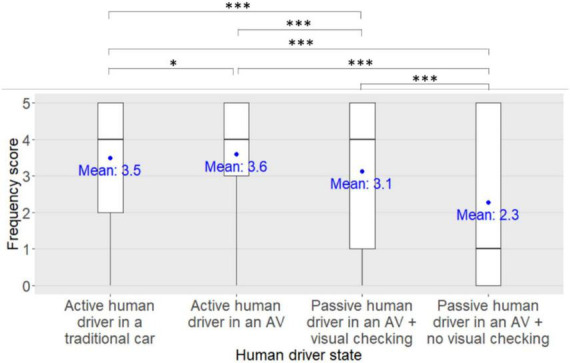
Main effect of the human driver state on the pedestrians’ frequency scores to ensure that the human driver has made eye contact in order to take an effective crossing decision. *: *pFDR* < 0.05, ***: *pFDR* < 0.001.

Regarding the pedestrians’ frequency scores to ensure that the human driver has made eye contact in order to take their effective crossing decision, a significant main effect of the human driver state was found [*F*_(3,1326)_ = 101.76, *p* < 0.001, η*^2^_*p*_* = 0.19, see Figure 4]. No significant main effect of the eHMI type [*F*_(4,442)_ = 1.46, *p* = 0.21, η*^2^_*p*_* = 0.01] nor of the participant’s age group [*F*_(3,442)_ = 0.45, *p* = 0.72, η*^2^_*p*_* < 0.01] were found. However, a significant human driver state x eHMI x participants’ age group interaction was found [*F*_(36,1326)_ = 2.18, *p* < 0.001, η*^2^_*p*_* = 0.06] although it was not supported by the *post-hoc* analyses (all *pFDRs* > 0.05). No other significant interactions between factors were found (all *ps* > 0.05).

*Post-hoc* analyses investigating the main effect of the human driver state on the participants’ frequency scores to ensure that the human driver has made eye contact in order to take their effective crossing decision revealed that the participants’ frequency scores were significantly lower when the human driver was passive in an AV with no visual checking on the road compared to when the human driver was passive in an AV with visual checking on the road (<0.001), and compared to when the human driver was driving in an AV (*pFDR* < 0.001) or a traditional car (*pFDR* < 0.001). In addition, the participants’ frequency scores were significantly lower when the human driver was passive in an AV with visual checking on the road compared to when the human driver was driving in an AV (*pFDR* < 0.001) or in a traditional car (*pFDR* < 0.001). Finally, the participants’ frequency scores were significantly lower when the human driver was driving in a traditional car compared to an AV (*pFDR* = 0.01).

### Conclusion of experiment A

This experiment aimed at investigating how pedestrians would like to visually communicate when encountering a human driver in an approaching car. We demonstrated that the human driver’s state inside the approaching car had an impact both on how pedestrians would express with their gaze their willingness to cross in front of the vehicle and on how they would take into consideration the human driver’s gaze in order to make their effective crossing decision. Indeed, results show that the more the human driver was described as passive in the driving task, the less the pedestrians reported wanting to visually communicate with the human driver. Moreover, when pedestrians had to take a crossing decision, we found that the characteristics of the approaching car modulated the frequency with which pedestrians ensured that the human driver had made eye contact. The AV led to a greater willingness to check for eye contact as compared with the conventional car when the pedestrians sought to take an effective crossing decision. However, the type of eHMI fitted on the approaching car was not shown in this experiment to influence visual communication between the pedestrians and the human driver in the approaching car. Lastly, no age-based effect was found, whether on how pedestrians would like to visually communicate their willingness to cross in front of the vehicle or how they would take the human driver’s gaze into consideration when deciding whether to cross.

## Experiment B: Cyclists

### Methods

#### Participants

Participants were recruited through social media and student networks. To be included in this survey questionnaire experiment, participants had to report cycling as their most frequent mode of travel. Hence, 279 French-speaking participants took part in the experiment (135 women and 144 men; mean age = 42.41 years old; SD of age = 16.21 years; age range = 15–77). Among them, 85.30% were living in town whereas the others were living in the countryside; 48.39% of the participants reported cycling every day, 42.29% reported cycling several times a week, while 6.45 and 2.87% reported cycling several times a month and several times a year, respectively.

#### Material

The questionnaire was identical to Experiment A, except that the four scenarios described situations of encounters between a cyclist and a human driver in an approaching car. Hence, participants were asked to imagine themselves as the cyclist in the described scenarios, located on a cycling path and with the intention to cross on the nearby crosswalk while a vehicle was approaching.

#### Procedure

The procedure was identical to that of Experiment A.

#### Factorial plan

##### Independent variables

The independent variables were identical to those of Experiment A except for the participants’ age groups. Participants were assigned to one of three age groups, namely, “cyclists under 26,” “26- to 45-year-old cyclists,” and “46- to 80-year-old cyclists,” according to their self-reported age. These age group intervals were defined with regard to French cyclists’ accidentology data (CEREMA, 2018, p. 12).

##### Dependent variables

The dependent variables were identical to those of Experiment A.

##### Operational hypotheses

The operational hypotheses were identical to those of Experiment A for H1, H2, and H3.

#### Statistical analyses

Statistical analyses were performed in a similar manner as in Experiment A.

### Results

#### Eye contact in order to express a willingness to cross

**FIGURE 5:**
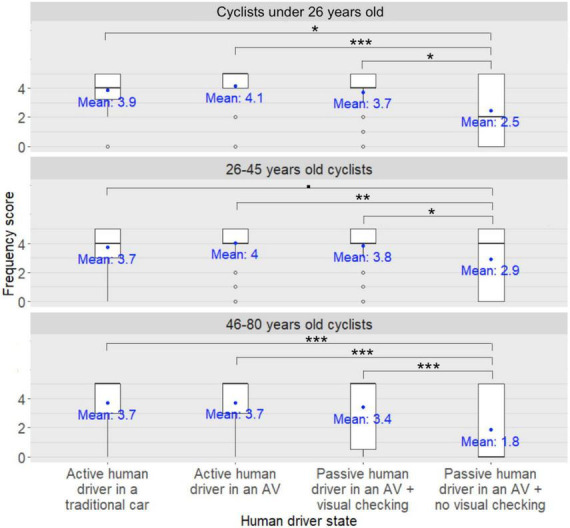
Human driver state × participant’s age group interaction on the cyclists’ frequency scores for seeking eye contact with the human driver of the approaching car in order to express their willingness to cross to the human driver ^•^: *pFDR* < 0.10, *: *pFDR* < 0.05, **: *pFDR* < 0.01, ***: *pFDR* < 0.001.

Regarding the cyclists’ frequency scores for seeking eye contact with the human driver of the approaching car in order to express their willingness to cross to the human driver, a significant main effect of the human driver state was found [*F*_(3,792)_ = 87.69, *p* < 0.001, η^2^*_*p*_* = 0.25]. No significant main effect of the eHMI type [*F*_(4,264)_ = 2.20, *p* = 0.07, η*^2^_*p*_* = 0.03] nor of the participant’s age group [*F*_(2,264)_ = 1.03, *p* = 0.38, η*^2^_*p*_* = 0.02] were found. However, a significant human driver state × participants’ age group interaction [*F*_(6,792)_ = 3.55, *p* = 0.002, η*^2^_*p*_* = 0.03, see Figure 5] was found as well as a significant human driver state x participants’ age group × eHMI interaction [*F*_(24,792)_ = 1.67, *p* = 0.05, η*^2^_*p*_* = 0.08], although the latter was not supported by *post-hoc* analyses (all *pFDRs* > 0.05). No other interactions between factors were found (all *ps* > 0.05).

*Post-hoc* analyses investigating the human driver state × participants’ age group interaction on the cyclists’ frequency scores for seeking eye contact with the human driver of the approaching car in order to express their willingness to cross to the human driver revealed that for three age groups (under 26, 26–45 years old, and 46–80 years old), the frequency scores were significantly lower when the human driver was passive in the AV with no visual checking on the road compared to when the human driver was passive in the AV with visual checking on the road (*pFDR* < 0.05, *pFDR* < 0.05, *pFDR* < 0.001). Moreover, the participants’ frequency scores were significantly lower when the human driver was passive in the AV with no visual checking on the road compared to when the human driver was driving in an AV (*pFDR* < 0.001, *pFDR* < 0.01, *pFDR* < 0.001, respectively). In addition, for two age groups (under 26, and 46–80 years old), the participants’ frequency scores were significantly lower when the human driver was passive in the AV with no visual checking on the road compared to when the human driver was driving in a traditional car (*pFDR* < 0.05, and *pFDR* < 0.001, respectively). Finally, for the 26- to 45-year-old participants, there was a trend for participants’ frequency scores to be significantly lower when the human driver was passive in the AV with no visual checking on the road compared to when the human driver was driving in a traditional car (*pFDR* = 0.06).

#### Eye contact in order to take an effective crossing decision

**FIGURE 6:**
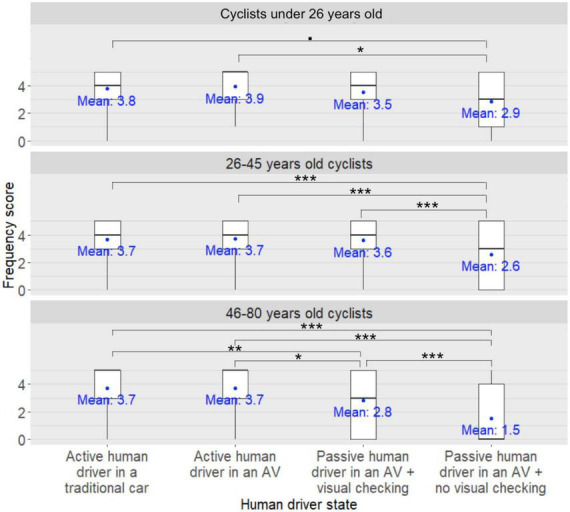
Human driver state × participant’s age group interaction on the cyclists’ frequency scores to ensure that the human driver has made eye contact in order to take their effective crossing decision ^•^: *pFDR* < 0.10, *: *pFDR* < 0.05, **: *pFDR* < 0.01, ***: *pFDR* < 0.001.

Regarding the cyclists’ frequency scores to ensure that the human driver has made eye contact in order to take their effective crossing decision, a significant main effect of the human driver state was found [*F*_(3,792)_ = 86.65, *p* < 0.001, η*^2^_*p*_* = 0.25], as well as a significant main effect of the participant’s age group [*F*_(2,264)_ = 3.57, *p* = 0.03, η*^2^_*p*_* = 0.03]. No main effect of the eHMI type [*F*_(4,264)_ = 1.26, *p* = 0.29, η*^2^_*p*_* = 0.02] was found but a significant human driver state × participants’ age group interaction [*F*_(6,792)_ = 6.22, *p* < 0.001, η*^2^_*p*_* = 0.04, see Figure 6] was found. Moreover, a significant human driver state × participants’ age group × eHMI interaction [*F*_(24,792)_ = 1.66, *p* = 0.02, η*^2^_*p*_* = 0.05] was found, although it was not supported by *post-hoc* analyses (all *pFDRs* > 0.05). No other interactions between factors were found (all *ps* > 0.05).

*Post-hoc* analyses investigating the main effect of the human driver state on the participants’ frequency scores to ensure that the human driver has made eye contact in order to take their effective crossing decision revealed that for participants aged under 26, 26–45, and 46–80 years, the frequency scores were significantly lower when the human driver was passive in the AV with no visual checking on the road compared to when the human driver was driving in an AV (*pFDR* = 0.02, *pFDR* < 0.001, *pFDR* < 0.001). In addition, for the 26–45-year-old and the 46–80-year-old participants, the frequency scores were significantly lower when the human driver was passive in the AV with no visual checking on the road compared to when the human driver was driving in a traditional car (*pFDR* = 0.001 and *pFDR* < 0.001, respectively). Similarly, there was a trend among those aged under 26 years for their frequency scores to be lower when the human driver was passive in the AV with no visual checking on the road compared to when the human driver was driving in a traditional car (*pFDR* = 0.08). Moreover, for the 26–45 and 46–80 year old participants, the frequency scores were significantly lower when the human driver was passive in the AV with no visual checking on the road compared to when the human driver was passive in the AV with visual checking on the road (*pFDR* < 0.001 and *pFDR* < 0.001, respectively). Finally, for the 46–80-year-old participants, the frequency scores were significantly lower when the human driver was passive in the AV with visual checking on the road compared to when the human driver was driving in an AV (*pFDR* = 0.011) or a traditional car (*pFDR* = 0.008).

### Conclusion of experiment B

This experiment aimed at investigating how cyclists would like to visually communicate when encountering a human driver in an approaching car. Results showed that the human driver’s state inside the approaching car had an impact both on how cyclists would express with their gaze their willingness to cross in front of the vehicle and on how they would take the human driver’s gaze into consideration when deciding whether to cross or not. Indeed, the cyclists reported a willingness to establish eye contact with the human driver of an approaching car to a lesser extent when the human driver was described as passive with no visual checking on the road compared to other human driver states, without any significant difference between the latter. However, when cyclists had to make a crossing decision, we found that with increasing age, the more passive the human driver was in the driving task the less the cyclists reported communicating by eye with the human driver. Lastly, the type of eHMI displayed on the approaching car was not shown in this experiment to influence visual communication between the cyclists and the human driver in the approaching car.

## Experiment C: Kick scooter users

### Methods

#### Participants

Participants were recruited through social media and student networks. To be included in this survey questionnaire experiment, participants had to report using a kick scooter as their most frequent mode of travel. Hence, 202 French-speaking participants took part in the experiment (91 women and 111 men; mean age = 35.81 years old; SD = 12.24 years; age range = 12–78). Among them, 82.67% were living in town whereas the others were living in the countryside; 27.23% of the participants reported using a kick scooter every day, 47.03% several times a week, and 21.785 and 3.96% several times a month and several times a year, respectively.

#### Material

The survey questionnaire experiment was identical to Experiment A, except that the four scenarios described situations of encounters between a kick scooter user and a human driver in an approaching car. Hence, participants were asked to imagine themselves as the kick scooter user in the described scenarios, located on a cycling path with the intention to cross on the nearby crosswalk while a vehicle was approaching.

#### Procedure

The procedure was identical to that of Experiment A.

#### Factorial plan

##### Independent variables

The independent variables were identical to those of Experiment A except for the participants’ age groups. Participants were assigned to one of four age groups, namely, “kick scooter users under 20,” “20–24 year-old kick scooter users,” “25–34 year-old kick scooter users,” and “kick scooter users 35 years old or more” according to their self-reported age. These age group intervals were defined with regard to French kick scooter users’ accidentology data (Bagou et al., 2021).

##### Dependent variable

The dependent variables were identical to those of Experiment A.

##### Operational hypotheses

The operational hypotheses were identical to those of Experiment A for H1, H2, and H3.

#### Statistical analyses

Statistical analyses were performed in a similar manner as in Experiment A.

### Results

#### Eye contact in order to express a willingness to cross

**FIGURE 7:**
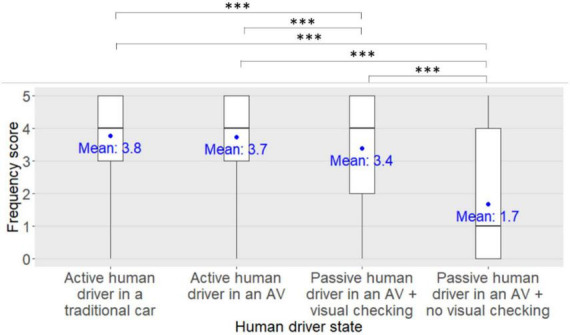
Main effect of the human driver state on the kick scooter users’ frequency scores for seeking eye contact with the driver of the approaching car in order to express their willingness to cross to the human driver. ***: *pFDR* < 0.001.

**FIGURE 8:**
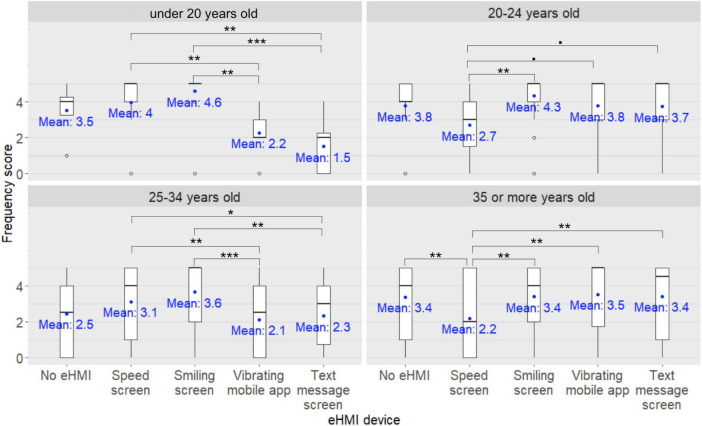
External Human-Machine Interface (eHMI) × participants’ age group interaction on the kick scooter users’ frequency scores for seeking eye contact with the human driver of the approaching car in order to express their willingness to cross to the human driver ^•^: *pFDR* < 0.10, *: *pFDR* < 0.05, **: *pFDR* < 0.01, ***: *pFDR* < 0.001.

Regarding the kick scooter users’ frequency scores for seeking eye contact with the human driver of the approaching car in order to express their willingness to cross to the human driver, significant main effects of the human driver state [*F*_(3,546)_ = 131.52, *p* < 0.001, η*^2^_*p*_* = 0.42, see Figure 7], of the eHMI type [*F*_(4,182)_ = 2.97, *p* = 0.02, η*^2^_*p*_* = 0.07], and of the participant’s age group [*F*_(3,182)_ = 5.26, *p* = 0.002, η*^2^_*p*_* = 0.08] were found. Moreover, a significant eHMI × participants’ age group interaction [*F*_(12,182)_ = 2.47, *p* = 0.005, η*^2^_*p*_* = 0.14, see Figure 8] was also found, as well as a significant human driver state × eHMI × participants’ age group interaction [*F*_(36,546)_ = 1.46, *p* = 0.04, η*^2^_*p*_* = 0.09], although the latter was not supported by *post-hoc* analyses (all *pFDRs* > 0.05). No other significant interactions between factors were found (all *ps* > 0.05).

*Post-hoc* analyses investigating the main effect of the human driver state on the participants’ frequency scores for seeking eye contact with the human driver of the approaching car in order to express their willingness to cross to the human driver revealed that the participants’ frequency scores were significantly lower when the human driver was passive in an AV with no visual checking on the road compared to when the human driver was passive in an AV with visual checking on the road (*pFDR* < 0.001), and compared to when the human driver was driving in an AV (*pFDR* < 0.001) or in a traditional car (*pFDR* < 0.001). Participants’ frequency scores were also significantly lower when the human driver was passive in an AV with visual checking on the road compared to when the human driver was driving in an AV (*pFDR* < 0.001) or in a traditional car (*pFDR* < 0.001). There was no difference in the participants’ frequency scores between when the human driver was driving in an AV and when the human driver was driving in a traditional car (*pFDR* = 0.58).

Furthermore, *post-hoc* analyses investigating the eHMI × participants’ age group interaction on the participants’ frequency scores for seeking eye contact with the human driver of the approaching car in order to express their willingness to cross to the human driver revealed that among the under-20s and the 25–34 year-old kick scooter users, the participants’ frequency scores were significantly lower when the participants were equipped with the vibrating mobile app compared to when the vehicle displayed the speed screen (*pFDR* = 0.009 and *pFDR* = 0.006, respectively) or the smiling screen (*pFDR* = 0.002 and *pFDR* < 0.001, respectively). Likewise, among the under-20s and the 25–34 year-old kick scooter users, the participants’ frequency scores were significantly lower when the vehicle was holding the text message screen compared to the speed screen (*pFDR* = 0.002 and *pFDR* = 0.048, respectively) or the smiling screen (*pFDR* < 0.001 and *pFDR* = 0.002, respectively). Among the 20–24 year-old and the over-35 kick scooter users, the participants’ frequency scores were significantly lower when the vehicle was holding the speed screen compared to the smiling screen (*pFDR* = 0.005 and *pFDR* = 0.006, respectively). Among the over-35 kick scooter users, the participants’ frequency scores were significantly lower when the vehicle was holding the speed screen compared to the text message screen (*pFDR* = 0.006), or no eHMI device (*pFDR* = 0.008), or compared when the participants were equipped with the vibrating mobile app (*pFDR* = 0.002). Lastly, among the 20–24 year-old kick scooter users, there was a trend for the participants’ frequency scores to be lower when the vehicle was holding the speed screen compared to the text message screen (*pFDR* = 0.051) and compared to when the participants were equipped with the vibrating mobile app (*pFDR* = 0.053).

#### Eye contact in order to take an effective crossing decision

**FIGURE 9:**
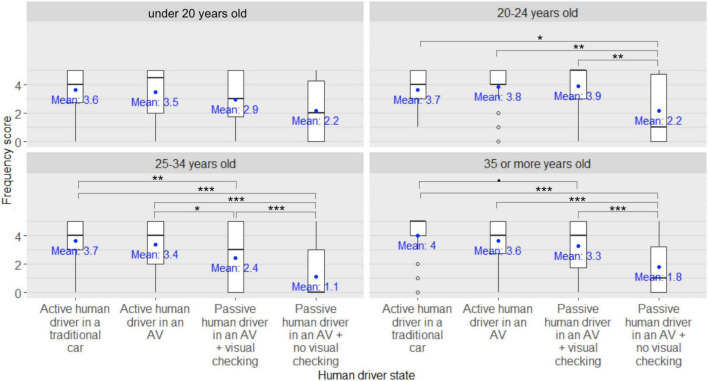
Human driver state × participants’ age group interaction on the kick scooter users’ frequency scores for ensuring that the human driver has made eye contact in order to take their effective crossing decision ^•^: *pFDR* < 0.10, *: *pFDR* < 0.05, **: *pFDR* < 0.01, ***: *pFDR* < 0.001.

**FIGURE 10:**
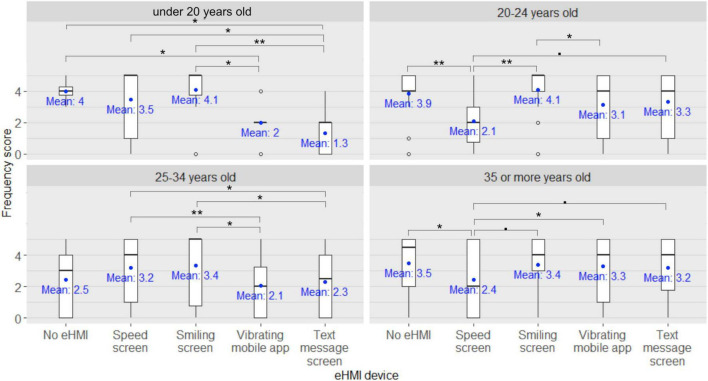
External Human-Machine Interface (eHMI) type × participants’ age group interaction on the kick scooter users’ frequency score for ensuring that the human driver has made eye contact in order to take their effective crossing decision ^•^: *pFDR* < 0.10, *: *pFDR* < 0.05, **: *pFDR* < 0.01.

Regarding the kick scooter users’ frequency scores for ensuring that the human driver has made eye contact in order to take their effective crossing decision, a significant main effect of the human driver state [*F*_(3,546)_ = 107.12, *p* < 0.001, η*^2^_*p*_* = 0.37], of the eHMI type [*F*_(4,182)_ = 3.41, *p* = 0.01, η*^2^_*p*_* = 0.07], and of the participants’ age group [*F*_(3,182)_ = 3.15, *p* = 0.03, η*^2^_*p*_* = 0.05] were found. A significant human driver state × participants’ age group interaction [*F*_(9,546)_ = 2.23, *p* = 0.02, η*^2^_*p*_* = 0.04, see Figure 9], as well as a significant eHMI type × participants’ age group interaction [*F*_(12,182)_ = 2.15, *p* = 0.02, η*^2^_*p*_* = 0.12, see Figure 10] were also found, and a significant human driver state × participants’ age group × eHMI interaction [*F*_(36,546)_ = 1.70, *p* = 0.01, η*^2^_*p*_* = 0.10] was found, although the latter was not supported by *post-hoc* analyses (all *pFDRs* > 0.05). The eHMI type × human driver state interaction did not reach significance [*F*_(12,546)_ = 1.56, *p* = 0.09, η*^2^_*p*_* = 0.04] (all *ps* > 0.05).

*Post-hoc* analyses investigating the eHMI × participants’ age group interaction on the participants’ frequency scores for ensuring that the human driver of the approaching car has made eye contact in order to take their effective crossing decision revealed no differences between the frequency scores among the under-20 kick scooter users with regards to the different human driver states (all *pFDRs* > 0.05). However, among the other three age groups of kick scooter users (20–24 years old, 25–34, and 35 or over), the participants’ frequency scores were lower when the human driver of the approaching car was passive in an AV with no visual checking on the road compared to when the human driver was passive in an AV with visual checking on the road (*pFDR* = 0.002, *pFDR* < 0.001, and *pFDR* < 0.001, respectively), or driving in an AV (*pFDR* = 0.006, *pFDR* < 0.001, and *pFDR* < 0.001, respectively) or in a traditional car (*pFDR* = 0.02, *pFDR* < 0.001, and *pFDR* < 0.001, respectively). In addition, among the 25–34 year-old kick scooter users, the participants’ frequency scores were lower when the human driver of the approaching car was passive in an AV with visual checking on the road compared to when the human driver was driving in an AV (*pFDR* = 0.02) or in a traditional car (*pFDR* = 0.002). Lastly, there was a trend among kick scooter users aged 35 years or over for participants’ frequency scores to be lower when the human driver of the approaching car was passive in an AV with visual checking on the road compared to when the human driver was driving a traditional car (*pFDR* = 0.06).

Furthermore, *post-hoc* analyses investigating the eHMI × participants’ age group interaction on the participants’ frequency scores for ensuring that the human driver of the approaching car has made eye contact in order to take their effective crossing decision revealed that among the under-20 kick scooter users, scores were lower when they were equipped with the vibrating mobile app compared to when the approaching car was holding the smiling screen (*pFDR* = 0.01), or no eHMI device (*pFDR* = 0.04). Likewise, their frequency scores were lower when the approaching car was holding the text message screen compared to the smiling screen (*pFDR* = 0.004), or the speed screen (*pFDR* = 0.02), or no eHMI device (*pFDR* = 0.02). Among the 20–24 year-old kick scooter users, participants’ frequency scores were lower when the approaching car was holding the speed screen compared to the smiling screen (*pFDR* = 0.004) or to no eHMI device (*pFDR* = 0.005), and when participants were equipped with the vibrating mobile app compared to when the approaching car was holding the smiling screen (*pFDR* = 0.04). In addition, there was a trend for their frequency scores to be lower when the approaching car was holding the speed screen compared to the text message screen (*pFDR* = 0.06). Among the 25–34 year-old kick scooter users, participants’ frequency scores were lower when they were equipped with the vibrating mobile app compared to when the approaching car displayed the smiling screen (*pFDR* = 0.01) or the speed screen (*pFDR* = 0.008). Similarly, their frequency scores were lower when the approaching car was equipped with the text message screen compared to the smiling screen (*pFDR* = 0.02) or the speed screen (*pFDR* = 0.04). Finally, among the over 35-year-old kick scooter users, the participants’ frequency scores were lower when the approaching car was holding the speed screen compared to no eHMI device (*pFDR* = 0.02) or to when the participants were equipped with the vibrating mobile app (*pFDR* = 0.046). There was a trend for their frequency scores to be lower when the approaching car was holding the speed screen compared to the smiling screen (*pFDR* = 0.054) or the text message screen (*pFDR* = 0.09).

### Conclusion of experiment C

This experiment aimed at investigating how kick scooter users would like to visually communicate when encountering a human driver in an approaching car. Our findings revealed that the more the human inside the approaching car was described as passive in the driving task, the less the kick scooter users reported willingness to establish eye contact with the human driver in order to express their willingness to cross. When scooter users had to decide whether to cross or not, the passiveness of the human driver inside the approaching car led also to a decline in the kick scooter users’ desire to consider eye contact, but this was observed only in individuals aged over 20. Furthermore, our results suggested that under certain circumstances, the presence of an eHMI signal modulated the way kick scooter users sought to visually communicate with a human driver inside an approaching car. Indeed, we found that among the under-20s and the 25–34-year-old kick scooter users, the fact of being equipped with a vibrating mobile app, as opposed to no eHMI device, sufficed to diminish the individuals’ desire to establish eye contact with the human driver in order to express their willingness to cross or to take their effective crossing decision. In addition, our results revealed a similar drop when the approaching car was described as wearing a text message eHMI that indicated explicitly whether to cross or not. By contrast, among the kick scooter users aged 20–24 and over 35, it was the presence of the speed screen eHMI on the approaching car, as opposed to no eHMI device, that lowered individuals’ willingness to establish eye contact with the human driver in order to express their desire to cross or to take their effective crossing decision.

## General discussion

Across three experiments investigating visual communication between vulnerable road users and human drivers, our findings highlighted that human driver disengagement in the driving task triggered a decline in vulnerable road users’ desire to communicate *via* eye contact (Experiments A–C). This was expected (H1) because navigating is a social activity (i.e., it involves several road users who cooperate in order to avoid traffic accidents) where eye contact plays a crucial role in the initiation and regulation of social encounters (Rakotonirainy et al., 2008; Jording et al., 2019). Hence, each road user must be aware of others in order to be able to receive and to send relevant cues. Experiment B revealed that the impact of the human driver disengagement in the driving task on visual communication was more pronounced in older age groups (H4). Moreover, we found in Experiment A that when the human driver was actively driving in the approaching car, the pedestrians’ reported desire to ensure that the human driver has made eye contact in order to take an effective decision to cross was higher when the vehicle was an AV compared to a traditional car. This could be due to the probable ambiguity of the situation. Indeed, in an AV, the drivenger is assumed to be relieved of the driving task. Yet, when the drivenger is driving the AV, there may be some doubt about who is in control inside the vehicle, leading vulnerable road users to seek more for eye contact with the human driver in order to assess his or her intentions when compared to a traditional car. Consistently, prior investigations reported that participants adopting a cyclist perspective in a scenario-based experiment behaved more cautiously when encountering an AV compared to a traditional car (Bazilinskyy et al., 2022). Yet, it is unclear why this was only observed in Experiment A (i.e., among pedestrians) when the participants aimed at taking their effective crossing decision. Ultimately, these findings lend weight to the importance of introducing new communication channels with the forthcoming arrival of AVs in road traffic in order to overcome the foreseeable pernicious changes they will bring (e.g., drivengers being out-of-the-loop in level-4 AVs). Moreover, engine noise may be absent in electrically powered vehicles such as AVs, with the result that the approach of the vehicle may not be heard by the surrounding road users. Hence, AVs must be able to communicate with the surrounding road users.

Interestingly, in Experiment A, no effect of the pedestrians’ age group was found on how they would like to visually communicate with drivengers. This may reflect attitudes that are deep-rooted at an early age when children are taught how to cross a road and that persist throughout their lifespan. In contrast, riding a bicycle or a kick scooter as modes of travel when crossing a road are skills that are less often taught to children, which may make riders’ attitudes more subject to age-related variations over the life course.

Critically, among kick scooter users demonstrated to be the most heavily involved in fatal injuries (Bagou et al., 2021), i.e., the under 20s and the 20–24 year-olds, some of the eHMI devices were shown to reduce their need for visual communication with the human driver of the approaching vehicle in Experiment C (H2 and H4). Specifically, the under-20 kick scooter users reported that they would consider eye contact when taking their effective crossing decision less often if the approaching car was holding the text message screen eHMI or if they were equipped with the vibrating mobile app, by contrast to no eHMI device. Because a text-based eHMI is regarded as the least ambiguous (Fridman et al., 2019), it is possible that the youngest individuals would preferentially rely on it in order to understand the approaching car’s intentions. Indeed, this type of signal is quick and easy to process, so they readily understand how to behave in front of the approaching car and may consider crossing without the need to strengthen their crossing decision with other clues. At the same time, the youngest kick scooter users may have also benefited from the signal of the vibrating mobile app because of a higher level of mobile media adoption. However, these kinds of signals could be dangerous in the event of system failure (e.g., the eHMI indicates to the facing vulnerable road users that they can cross when in fact the vehicle does not give way) and road users’ overtrust in the system, namely, a level of trust that exceeds the system capabilities (Lee and See, 2004). For example, it has been shown that some pedestrians were likely to rely on the explicit crossing advice supplied by a light band eHMI, ignoring the inconsistent vehicle kinematic information during the street crossing (Kaleefathullah et al., 2020). Hence, the consequences for safety may be dramatic if vulnerable road users blindly follow eHMI crossing advice.

On the other hand, our results also showed that the 20–24-year-old kick scooter users reported that they would use the eye contact cue less often if the approaching car was holding the speed screen eHMI compared to no eHMI device, particularly when they aimed to take an effective crossing decision. Consistent with that, there has been evidence that the vehicle’s speed is one of the most important pieces of information that enters into consideration in vulnerable road users’ crossing decision-making process (Clamann et al., 2017; Sucha et al., 2017; Palmeiro et al., 2018; Dey et al., 2021; Lee et al., 2021). Hence, giving this piece of information in a tangible way before the approaching car comes close to the kick scooter users could help them to enter the road earlier with less need to visually communicate. It should be noted that although Experiment A did not succeed in showing any benefit of the speed screen eHMI among pedestrians, other work using more immersive scenarios with video clips of real-traffic scenes has suggested that such a display could reduce the pedestrians’ time to collision with an approaching car (Sahaï et al., 2021). In a nutshell, such a display may promote traffic fluency by contrast to single-user-targeted crossing advice. Moreover, this kind of signal engages vulnerable road users’ thinking skills, considering them as active information processing agents rather than as passive individuals who merely follow an indication, thus enabling them to remain active agents in the road ecosystem.

Finally, it could be said that deciding to cross in front of a car solely on the basis of the information provided by an eHMI, and without feeling the need to communicate with the human driver, may reflect a high level of trust toward this type of communication system. Indeed, prior research focusing on pedestrians reported a negative correlation between the time spent checking for the AV while crossing and the self-reported trust in AV (Jayaraman et al., 2019). But as mentioned earlier, kick scooter users were likely to trust certain eHMI device types rather than others depending on their age. Yet, the issue of finding the eHMI signal to deliver that suits all road users, given their travel habits and their individual characteristics, remains still open to debate. Nevertheless, these three crowdsourced experiments have highlighted vulnerable road users’ bottom-up expectations about future mobility systems. In particular, while studies reported benefits of eHMI signals in the form of crossing advices on pedestrians (e.g., Cœugnet et al., 2017; Dey et al., 2021), we claim that an eHMI signal displaying the actual vehicle speed could be beneficial to 20–24-year-old kick scooter users.

## Data availability statement

The raw data supporting the conclusions of this article will be made available by the authors, without undue reservation.

## Ethics statement

The studies involving human participants were reviewed and approved by the Comité d’Éthique de la Recherche–CER, Université Fédérale Toulouse Midi-Pyrénées. Written informed consent to participate in this study was provided by the participants’ legal guardian/next of kin.

## Author contributions

AS, EL, LC, and CL contributed to design the study. AS implemented the three experiments. AS and CL were responsible for the recruitment of the participants. AS performed the pre-processing and the statistical analyses of the collected data in concert with EL, LC, and CL. AS provided the interpretations of the data and wrote the manuscript with input from EL, LC, and CL. All authors contributed to the article and approved the submitted version.
