# Supplementary material for: Crossing the street in front of an autonomous vehicle: An investigation of eye contact between drivengers and vulnerable road users
Source: Front Psychol. 2022 Oct 28;13:981666. doi: 10.3389/fpsyg.2022.981666 (PMC9649438; doi:10.3389/fpsyg.2022.981666)
Supplement: Supplementary file 1 [file Presentation_1.pdf]

Appendix A: exhaustive description of the four scenarios used in the experiment

Scenario 1:

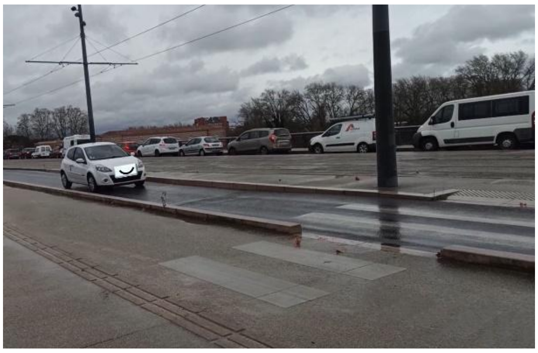

Imagine yourself walking on the sidewalk, near a crosswalk that allows you to cross a single lane. There are no traffic lights (e.g. traffic lights, pedestrian lights). You want to cross the lane on the crosswalk while a traditional car is approaching.

This car has been equipped with an external screen that displays the speed of the car in real time/a smile when the human driver is about to yield otherwise a horizontal bar /a text message indicating “you can cross” when the human driver is about to yield otherwise “you cannot cross”.

OR

You are equipped with a vibrating mobile app that sends you a vibration when the human driver is about to yield.

Here is a picture of the device (e.g., the smiling screen):

*See **Figure 1** for the pictures of the other types of eHMI displays.*

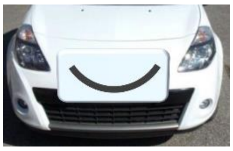

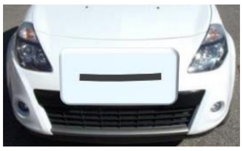

In this situation, how often would you seek eye contact with the human driver in order to express your willingness to cross?

O never O rarely O from time to time O sometimes O often O always

In this situation, how often would you ensure that the driver has made eye contact in order to make the effective decision to cross?

O never O rarely O from time to time O sometimes O often O always

Scenario 2:

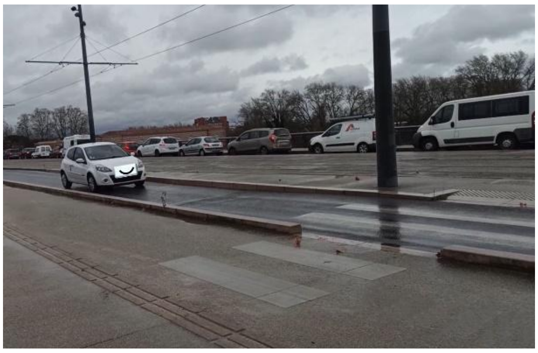

Imagine yourself walking on the sidewalk, near a crosswalk that allows you to cross a single lane. There are no traffic lights (e.g. traffic lights, pedestrian lights). You want to cross the lane on the crosswalk while an autonomous car is approaching. The autonomous car is able to manage the different aspects of driving (e.g. lane keeping, acceleration/braking, obstacle detection) without the human driver inside having to supervise the driving when the autonomous mode is activated.

This car has been equipped with an external screen that displays the speed of the car in real time/a smile when the car is about to yield otherwise a horizontal bar /a text message indicating “you can cross” when the car is about to yield otherwise “you cannot cross”.

OR

You are equipped with a vibrating mobile app that sends you a vibration when the car is about to yield.

Here is a picture of the device (e.g., the smiling screen):

*See **Figure 1** for the pictures of the other types of eHMI displays.*

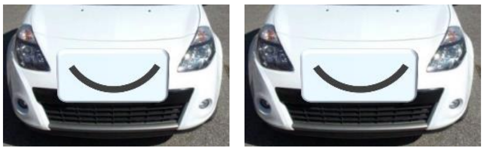

However, in this situation, it is the human driver who drives the approaching car: s/he has her/his hands on the wheel and looks at the road.

In this situation, how often would you seek eye contact with the human driver in order to express your willingness to cross?

O never O rarely O from time to time O sometimes O often O always

In this situation, how often would you ensure that the driver has made eye contact in order to make the effective decision to cross?

O never O rarely O from time to time O sometimes O often O always

Scenario 3:

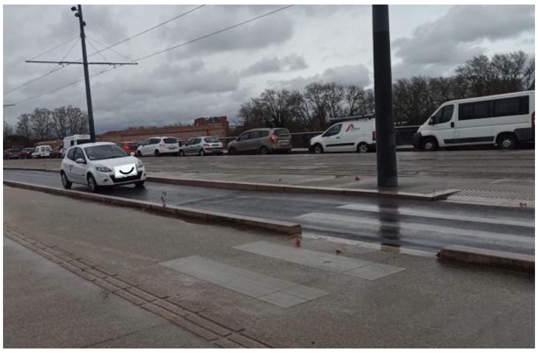

Imagine yourself walking on the sidewalk, near a crosswalk that allows you to cross a single lane. There are no traffic lights (e.g. traffic lights, pedestrian lights). You want to cross the lane on the crosswalk while an autonomous car is approaching. The autonomous car is able to manage the different aspects of driving (e.g. lane keeping, acceleration/braking, obstacle detection) without the human driver inside having to supervise the driving when the autonomous mode is activated.

This car has been equipped with an external screen that displays the speed of the car in real time/a smile when the car is about to yield otherwise a horizontal bar /a text message indicating “you can cross” when the car is about to yield otherwise “you cannot cross”.

OR

You are equipped with a vibrating mobile app that sends you a vibration when the car is about to yield.

Here is a picture of the device (e.g., the smiling screen):

*See **Figure 1** for the pictures of the other types of eHMI displays.*

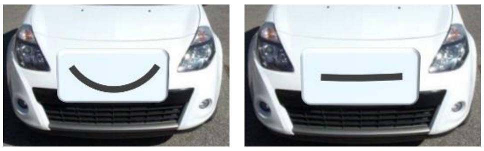

In this situation, it is the autonomous car that drives: the human driver does not drive. Nevertheless, the human driver keeps his eyes on the road.

In this situation, how often would you seek eye contact with the human driver in order to express your willingness to cross?

O never O rarely O from time to time O sometimes O often O always

In this situation, how often would you ensure that the driver has made eye contact in order to make the effective decision to cross?

O never O rarely O from time to time O sometimes O often O always

Scenario 4:

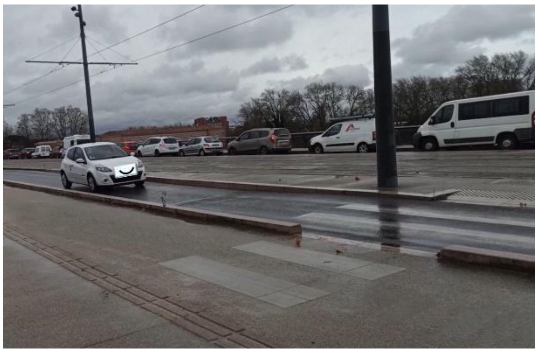

Imagine yourself walking on the sidewalk, near a crosswalk that allows you to cross a single lane. There are no traffic lights (e.g. traffic lights, pedestrian lights). You want to cross the lane on the crosswalk while an autonomous car is approaching. The autonomous car is able to manage the different aspects of driving (e.g. lane keeping, acceleration/braking, obstacle detection) without the human driver inside having to supervise the driving when the autonomous mode is activated.

This car has been equipped with an external screen that displays the speed of the car in real time/a smile when the car is about to yield otherwise a horizontal bar /a text message indicating “you can cross” when the car is about to yield otherwise “you cannot cross”.

OR

You are equipped with a vibrating mobile app that sends you a vibration when the car is about to yield.

Here is a picture of the device (e.g., the smiling screen):

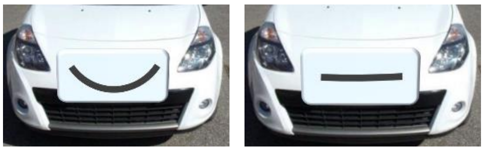

In this situation, it is the autonomous car that drives: the human driver does not drive. Moreover, the human driver does not look at the road.

In this situation, how often would you seek eye contact with the human driver in order to express your willingness to cross?

O never O rarely O from time to time O sometimes O often O always

In this situation, how often would you ensure that the driver has made eye contact in order to make the effective decision to cross?

O never O rarely O from time to time O sometimes O often O always
